# Evaluating Imputation Algorithms for Low-Depth Genotyping-By-Sequencing (GBS) Data

**DOI:** 10.1371/journal.pone.0160733

**Published:** 2016-08-18

**Authors:** Ariel W. Chan, Martha T. Hamblin, Jean-Luc Jannink

**Affiliations:** 1 Section of Plant Breeding and Genetics, School of Integrative Plant Sciences, Cornell University, Ithaca, NY, United States of America; 2 Institute for Genomic Diversity, Cornell University, Ithaca, NY, United States of America; 3 RW Holley Center for Agriculture and Health, United States Department of Agriculture—Agricultural Research Service, Ithaca, NY, United States of America; Clemson University, UNITED STATES

## Abstract

Well-powered genomic studies require genome-wide marker coverage across many individuals. For non-model species with few genomic resources, high-throughput sequencing (HTS) methods, such as Genotyping-By-Sequencing (GBS), offer an inexpensive alternative to array-based genotyping. Although affordable, datasets derived from HTS methods suffer from sequencing error, alignment errors, and missing data, all of which introduce noise and uncertainty to variant discovery and genotype calling. Under such circumstances, meaningful analysis of the data is difficult. Our primary interest lies in the issue of how one can accurately infer or impute missing genotypes in HTS-derived datasets. Many of the existing genotype imputation algorithms and software packages were primarily developed by and optimized for the human genetics community, a field where a complete and accurate reference genome has been constructed and SNP arrays have, in large part, been the common genotyping platform. We set out to answer two questions: 1) can we use existing imputation methods developed by the human genetics community to impute missing genotypes in datasets derived from non-human species and 2) are these methods, which were developed and optimized to impute ascertained variants, amenable for imputation of missing genotypes at HTS-derived variants? We selected Beagle v.4, a widely used algorithm within the human genetics community with reportedly high accuracy, to serve as our imputation contender. We performed a series of cross-validation experiments, using GBS data collected from the species *Manihot esculenta* by the Next Generation (NEXTGEN) Cassava Breeding Project. NEXTGEN currently imputes missing genotypes in their datasets using a LASSO-penalized, linear regression method (denoted ‘glmnet’). We selected glmnet to serve as a benchmark imputation method for this reason. We obtained estimates of imputation accuracy by masking a subset of observed genotypes, imputing, and calculating the sample Pearson correlation between observed and imputed genotype dosages at the site and individual level; computation time served as a second metric for comparison. We then set out to examine factors affecting imputation accuracy, such as levels of missing data, read depth, minor allele frequency (MAF), and reference panel composition.

## Introduction

Well-powered genomic studies require genome-wide marker coverage across many individuals. Many genotyping methods exist, and one typically selects a genotyping platform based on budgetary constraints and the available molecular tools for the species in question. Genetic variation in the human genome, for instance, has largely been captured using single-nucleotide polymorphism (SNP) arrays that can assay up to 2.5 million variants [1]. The per-sample and array-design costs of these assays, however, make them accessible only to well-funded model systems. For species lacking a complete reference genome or predesigned high-density SNP genotyping arrays, high-throughput sequencing (HTS) methods, such as Genotyping-By-Sequencing (GBS), offer an economic approach for surveying variants at the genome level. The multiplex capabilities of HTS methods allow for great flexibility in experimental design. For instance, given a fixed number of sequencing reads and genome size, one can choose to sequence a small number of individuals, allocating the reads among a small number of individuals, or one can choose to distribute the reads among a larger sample of individuals. The former framework generates datasets with relatively low levels of missing data. The small sample size limits the number of detected variants, but this may be a moot point depending on the biological question one wishes to address. For studies requiring large sample sizes and dense genome-wide marker coverage, e.g. genome-wide association studies (GWAS) and genomic selection (GS), the latter genotyping framework is preferable, and one can impute or infer missing genotypes with appropriate imputation methods [2].

Genotype imputation is a well-established statistical technique for estimating unobserved genotypes. Many genotype imputation algorithms and software packages exist, but most were primarily developed by and optimized for the human genetics community, a field where a complete and accurate reference genome has been constructed and SNP arrays have, in large part, been the common genotyping platform. These algorithms differ in their details but all essentially pool information across individuals in either a study sample or a reference panel or both to estimate haplotype frequencies from the observed genotype data, imputing missing genotypes simultaneously. Although the statistical methods for genotype imputation are now highly developed and widely used, selecting the set of haplotypes to include in the reference panel for maximum imputation accuracy in a given study population remains unclear. Selection schemes typically take one of two approaches: a ‘best match’ approach, which attempts to construct a reference panel that closely matches the ancestry of the study sample, or a ‘cosmopolitan’ approach, which makes use of all available haplotypes [3].

To assess the applicability of human-tailored imputation algorithms in non-model species datasets, we evaluated the imputation performance of Beagle v.4, a widely used haplotype-phasing algorithm with reportedly high accuracy, in low-depth GBS-generated data collected from the species *Manihot esculenta* (commonly referred to by its colloquial name ‘cassava’). We compared Beagle v.4 to a LASSO-penalized, linear regression imputation method (denoted glmnet). We chose Beagle v.4 over other haplotype-phasing programs because the algorithm 1) scales well to large sample sizes (>1000) while other algorithms require some form of parameter space reduction to be computationally competitive, 2) requires no parameter specification, e.g. effective population size, 3) takes genotype likelihoods as input, and 4) performs genotype calling [4]. The Next Generation (NEXTGEN) Cassava Breeding Project currently employs glmnet to impute missing genotypes in NEXTGEN datasets; we selected glmnet to serve as a benchmark method for this reason. Glmnet takes a linear regression approach to genotype imputation. The algorithm assumes that any locus on a given chromosome can be modeled as a linear combination of other intra-chromosomal loci, independent of locus distance and locus order. Such methods model only the statistical correlations between loci and make no attempts at relating observed correlations to underlying biological phenomena, such as linkage disequilibrium (LD; the nonrandom association of alleles among linked loci). Results from [5] show that imputation of unordered markers can be accurate, particularly when LD between markers is high and when individuals in the study sample share recent common ancestry.

We evaluated Beagle and glmnet under three imputation scenarios: imputation guided by 1) no reference panel, 2) a reference panel with large genetic diversity (reference panel A), and 3) a reference panel that closely matches the ancestry of the study sample (reference panel B). We describe the composition of reference panel A and B in greater detail in the Methods and Materials section. We provide a schematic drawing of reference panel A and B in S1A Fig and of the three imputation scenarios in S1B Fig. We performed a series of cross-validation experiments using GBS data collected from the species *Manihot esculenta* by NEXTGEN. For simplicity, we focused on the situation where the reference haplotypes in scenario 2 and 3 are defined on the same set of polymorphic sites as those found in the study sample. For each cross-validation experiment, we measured imputation accuracy at both the site- and individual-level, using the sample Pearson correlation statistic as an estimate of accuracy. We assessed the impact of missing data, read depth, minor allele frequency (MAF), and reference panel composition on imputation accuracy. We report the computation requirement and a scalar summary of imputation accuracy measured at the site and individual for Beagle and glmnet under each scenario.

## Materials and Methods

We evaluated the performance of Beagle and glmnet under three imputation scenarios using data collected at biallelic SNPs on chromosome 5 from two NEXTGEN cassava populations: the International Institute of Tropical Agriculture’s (IITA) Genetic Gain (GG) population, a collection of historically important clones, and IITA’s Cycle 1 (C1) population. We first describe how the sequence data was generated and processed then provide a description of the two IITA populations.

### Data generation and variant calling

*Ape*KI GBS libraries were constructed at the Institute for Genomic Diversity at Cornell University and sequenced on the Illumina HiSeq 2000/2500 at the Biotechnology Resource Center at Cornell University following the protocol outlined in [6]. Converting the raw read data into a final set of SNP calls involved a number of steps; a complete description of the protocol is beyond the scope of this paper. We refer the reader to [7] and https://bitbucket.org/tasseladmin/tassel-5-source/wiki/Tassel5GBSv2Pipeline for a detailed description of version 4 and 5 of the TASSEL-GBS bioinformatics pipeline, respectively. SNPs were extracted from the raw sequence data using the TASSEL 5.0 GBS discovery pipeline with alignment to the *Manihot esculenta* v.6 assembly. Sequence reads generated by GBS assays were trimmed or padded to 64 bases and subjected to quality filters (refer to section ‘Favoring allelic redundancy over quality scores’ of [7]). The filtered sequence reads were aligned to the cassava reference genome version 6 assembly. Genotype calling then proceeded for each individual by counting the number of times each allele was observed and using empirically determined thresholds for genotype calls. SNP calling was then performed using the inferred genotypes. To minimize ascertainment bias, all NEXTGEN samples (in addition to non NEXTGEN samples) sequenced to date were used for variant detection. Putative SNPs were filtered based on a minimum minor allele frequency (mnMAF) of 0.001. NEXTGEN opted to use a relatively low-stringency filter since false-positive variants can be filtered out in subsequent steps. We obtained 18 VCF files (one VCF file per chromosome) after processing the raw GBS sequence reads from NEXTGEN samples. The raw VCF file for chromosome 5, a chromosome approximately 30 Mbp in length, contained 30018 entries (variant sites) and 15750 samples, 164 of which were blank negative controls. S2 Fig shows the distribution of variants across the length of chromosome 5. As of writing this manuscript, the data we analyzed are free and publically available at www.cassavabase.org.

Each sample ID (i.e. column name) in the VCF files follows the following format: ‘ShortName:LibraryPrepID’. Upon closer examination, we found 554 ‘ShortNames’ that appear >2 times in the VCF file for chromosome 5. Samples sharing an identical ‘ShortName’ represent (supposed) technical or biological replicates of a unique individual. Before merging the sequence data from samples sharing an identical ‘ShortName’, we applied an Expectation-Maximization (EM) algorithm to detect mislabeling of samples among technical and biological replicates (unpublished). We merged the sequence data for cases where the algorithm detected no error. We then removed non-biallelic sites from the dataset, leaving a total of 20302 biallelic SNPs for analysis. S2 Fig shows the distribution of biallelic SNPs across the length of chromosome 5.

The FORMAT field of the VCF file consists of five colon-separated, sub-fields: genotype (GT), allelic read depth (AD), read depth (DP), genotype quality (GQ), and Phred-scaled likelihood (PL). For our purposes, we were interested in only the AD subfield, which encodes the observed counts of each of the two alleles in individual *d* at site *v*: $X_{d}^{\left(v\right)}=\left(N_{A}^{\left(v,d\right)},N_{B}^{\left(v,d\right)}\right)$, where $N_{A}^{\left(v,d\right)}$ and $N_{B}^{\left(v,d\right)}$ denote the observed counts of allele A and allele B, respectively, in individual *d* at site *v*. To ensure that genotype likelihoods were calculated in a consistent manner, we computed genotype likelihoods for each individual at each site using the data stored in the AD subfield rather than using those provided in the PL subfield of the VCF file. Given observed data $X_{d}^{\left(v\right)}$ and fixed sequencing error rate *e* = 0.01, we computed the likelihood for genotype $G_{d}^{\left(v\right)}=g$. We calculated genotype likelihoods for a single individual at a single site independent of all other individuals and sites in the sample using the following equation:

$$P\left(X_{d}^{\left(v\right)}|G_{d}^{\left(v\right)}=g,e\right)=\left(\begin{array}{c} N_{A}^{\left(v,d\right)}+N_{B}^{\left(v,d\right)}\\ N_{B}^{\left(v,d\right)} \end{array}\right){\left(1-p_{B}\right)}^{N_{A}^{\left(v,d\right)}}{{(p}_{B})}^{N_{B}^{\left(v,d\right)}}$$

$$p_{B}=\left\{ \begin{array}{c} e,\\ 0.50,\\ 1-e, \end{array}\;\begin{array}{c} \text{when}\\ \text{when}\\ \text{when} \end{array}\right.\;\begin{array}{c} g=AA\\ g=AB\\ g=BB \end{array}.$$

We estimated posterior probabilities for the three genotypes using the likelihoods defined above and assuming a uniform genotype prior. We summarized posterior probabilities into genotype dosages since the glmnet algorithm can only take scalar-valued genotypes as input. Genotype dosages take values in [0,2] or NA for the case where no data is observed for a given individual at a site. We converted genotype likelihoods into normalized, Phred-scaled likelihoods to use as input for Beagle.

### Germplasm

IITA has a large GG population for which there are many years of historical phenotype data collected in many environments. NEXTGEN selected a subset of GG individuals to serve as a training population (TP) for genomic selection (GS) at IITA. NEXTGEN selected an individual if plant material still existed for the individual (i.e. DNA could be extracted to obtain genotype data) and if phenotype records for the individual were based on a sufficient number of observations. As of writing this report, 694 individuals met these criteria [8]. From this point forward, we refer to these 694 individuals as the GG population. GG individuals are listed in S1 Table. Genomic estimated breeding values (GEBVs) were obtained using the genomic best linear unbiased prediction (BLUP) method and the top GG individuals were selected to serve as founders of the IITA GS breeding program. To avoid inbreeding depression, NEXTGEN designed a crossing framework based on results from a k-means clustering analysis, crossing two GG individuals only if they belonged to different clusters. Based on pedigree records (refer to S2 Table), a total of *y* ≥474 crosses were made, with only a subset of these crosses (134 crosses using 82 individuals) producing viable progeny. The large variation in viable progeny number among attempted crosses results from the wide variation in flowering time, rate, and fertility in cassava [9]. Viable progeny from GG crosses collectively form the C1 population. Two randomly sampled individuals from the C1 population are nominally related in one of three possible ways: the two individuals are 1) full siblings, 2) half siblings, or 3) unrelated. We have pedigree records for 2207 C1 individuals but found 2490 individuals in the VCF file whose sample IDs indicate C1 population membership (i.e. samples with sample name prefix “2013_” and “TMS13”). We used all 2490 C1 individuals as the target of imputation for scenarios 2 and 3.

Inconsistencies among sources of information (i.e. the pedigree record, the sequence data in the VCF file, and the list of 694 GG individuals) influenced the design of the two reference panels used in imputation scenarios 2 and 3. According to the pedigree record, 82 individuals gave rise to the C1 population; however, only 78 of these 82 supposed C1 parents appear in the list of 694 GG individuals. We expected all C1 parents to appear in the list of GG individuals. We found sequence data for these 78 individuals in the VCF file. Of the remaining four individuals listed as C1 parents in the pedigree record, we found sequence data for only two individuals B9200061 and B9200068 in the VCF file. We expected all C1 parents to have sequence data since this information was required for estimation of breeding values. We found no sequence data for individuals I970466 and I974769 in the VCF file.

The 694 GG individuals served as the reference panel for scenario 2 (reference panel A, representing a “cosmopolitan” reference panel). The 80 individuals listed as C1 parents in the pedigree record for whom we have sequence data served as the reference panel for scenario 3 (reference panel B, representing a “best-match” reference panel). The intersection of reference panel A and panel B consists of 78 C1 parents. We provide a schematic drawing of reference panel A and B in S1A Fig.

The two reference panels collectively contain 696 unique individuals (the union of reference panel A and panel B). We performed a principal component analysis (PCA) to explore whether there is any evidence of population structure among the 696 reference panel individuals. We calculated the realized additive relationship matrix for the 696 reference panel individuals at a subset of the 20205 biallelic SNPs using the function “A.mat” from the R package “rrBLUP” [10], [11]. We excluded sites with >50% missing data (max.missing = 0.5) from the calculation and imputed missing dosage values using the “EM” option (impute.method =“EM”). We then performed PCA through eigenvalue decomposition of the realized additive relationship matrix (covariance matrix) using the R function “prcomp” and plotted the first two principal components (S3 Fig). We observed little evidence of subpopulation structure among the 696 reference panel individuals.

### Dataset for scenario 1 (imputation using no reference)

If each individual and each site in the study sample have a low proportion of missing data, no reference panel is needed to impute the missing genotypes in the sample; the almost complete data from the other individuals and the high marker density should provide sufficient information to impute with high accuracy. We tested this concept using the 694 GG individuals as our study sample. We extracted the genotype dosages and normalized, Phred-scaled likelihoods for the GG individuals at biallelic sites (*n* = 20302). S4 Fig shows the distribution of the proportion of missing data per site. The term “missing” denotes zero reads observed at a given site for a given individual. We removed sites with >90% missing data, leaving a total of 20205 sites for cross-validation experiment 1. We use this same set of sites for imputation scenario 2 and 3 for reasons given in the proceeding section. S5A and S5B Fig show the distribution of the mean read depth per site averaged across all 694 GG individuals and across all 696 reference panel individuals, respectively.

### Datasets for scenario 2 and 3

We assessed the impact of reference panel composition on imputation accuracy using C1 individuals (*n* = 2490) as the target of imputation. We constructed two reference panels, one designed to represent a cosmopolitan reference panel for imputation scenario 2 and the other designed to represent a best-match reference panel for scenario 3. Variants absent from the reference panel, but present in the study sample, cannot be imputed. We, therefore, focused on the situation where the reference panel is defined on the same set of polymorphic sites as those found in the study sample, using the same set of 20205 biallelic SNPs defined in scenario 1.

We extracted genotype dosages and normalized, Phred-scaled likelihoods for the 2490 C1 individuals. To construct the reference panels for scenario 2 and 3, which collectively consist of 696 individuals, we extracted genotype dosages and normalized, Phred-scaled likelihoods for the 696 reference panel individuals. We ran the glmnet and Beagle imputation algorithms, using the extracted genotype dosages and normalize, Phred-scaled likelihoods for the 696 individuals as input, respectively. We constructed the cosmopolitan reference panel for Beagle (glmnet) using the inferred haplotypes (imputed genotype dosages) from the 694 GG individuals; we constructed the best-match reference panel for Beagle (glmnet) using the inferred haplotypes (imputed genotype dosages) from the 80 C1 parents. Although a reference panel cannot be explicitly specified when imputing with glmnet, the algorithm can still make use of the information encoded in non-study sample individuals. The increased sample size of the training data should, in theory, increase imputation accuracy.

### Glmnet Algorithm

We used the R package glmnet to fit a LASSO-penalized, linear regression model to the observed genotype data [12]. The glmnet imputation algorithm described here employs a combination of both variable selection and the least absolute angle and selection operator (LASSO). LASSO penalized estimates are solutions to an optimization problem of the form:

$$\tilde{\beta }={argmin}_{\beta }\;\left\{\sum_{i=1}^{N}{\left(y_{i}-\beta_{0}-\sum_{j=1}^{p}x_{ij}\beta_{j}\right)}^{2}+\lambda \sum_{j=1}^{p}{\left|\beta_{j}\right|}^{q}\right\}.$$

We set *q* = 1. The variable *λ* is a regularization parameter that controls the trade-offs between lack of fit and model complexity; *λ* ≥ 0 [13]. In addition to shrinking estimates toward zero, LASSO can perform variable selection, setting a subset of regression coefficients to zero [13]. The algorithm initializes by imputing missing genotypes at site *v* to the mean genotype at site *v*. Although the LASSO performs variable selection on its own, we performed an initial round of variable selection to decrease computation time—shrinking the variable search space to a subset of 60 markers rather than using all markers on a chromosome as potential predictors of genotype. We calculated pairwise correlations between the target marker and all intra-chromosomal markers, retaining the 60 markers that showed the strongest correlation with the target marker. We selected a maximum retention number of 60 arbitrarily. Other approaches for shrinking the variable search space exist but were not explored in this study. By default, glmnet selects a lambda value using 10-fold cross-validation, looking at 100 different lambda penalty coefficients. To decrease computation time, 5-fold cross validation was performed on 10 lambda values.

### Beagle v.4

Beagle v.4 is an iterative algorithm for fitting a local haplotype hidden Markov model (HMM) to genotype data. The algorithm alternates between model building and sampling, using stochastic expectation maximization (EM) to converge towards the most probable solutions [14]. There are five components to an HMM: 1) hidden states, 2) observed values, 3) state-transition probabilities, 4) emission probabilities, and 5) initial-state probabilities [15]. The underlying hidden states of an HMM generate the observed data, and the state-transition probabilities, emission probabilities, and initial-state probabilities are parameters of the HMM. In the context of haplotype phase and missing genotype inference, the observed data are the unphased genotypes, while the hidden states represent haplotype membership and the true, underlying genotypes. Beagle estimates state-transition probabilities, emission probabilities, and initial-state probabilities from the data.

The algorithm begins by imputing missing genotypes according to allele frequencies and randomly phasing heterozygous genotypes. Beagle v.4 then uses these initial haplotype estimates to obtain estimates of the HMM parameters. The algorithm constructs a directed acyclic graph (DAG) using the haplotype data and estimates the HMM parameters using observed haplotype counts and the assumption of Hardy-Weinberg Equilibrium (HWE). [16]. Browning provides a detailed explanation of how the algorithm constructs the graphical model in [16]. After constructing the model, Beagle samples four pairs of haplotypes per individual from the posterior distribution of haplotypes conditioned on the observed genotypes. These sampled haplotypes serve as input for the next iteration to re-estimate the model parameters. The model building and sampling procedure repeats for five burn-in iterations, followed by an additional five iterations. Beagle v.4 outputs a consensus haplotype for each individual, which is constructed from the 20 haplotypes sampled during the non burn-in iterations. In addition to consensus haplotypes, Beagle v.4 outputs imputed genotype dosages (also known as posterior mean genotypes) for each individual at each site. A reference panel can be specified in Beagle v.4 with the *ref* parameter. All genotypes in the reference panel must be non-missing and phased.

### Measuring imputation accuracy

There are various metrics of imputation accuracy: imputation correlation, the Pearson correlation between observed and imputed genotypes, imputation concordance, the proportion of correctly imputed genotypes, imputation quality score (IQS), the concordance adjusted for chance agreement), etc. [17]. We selected the Pearson correlation coefficient to serve as our metric of imputation accuracy at the site level since its interpretation does not depend on MAF. The sample Pearson correlation between two variables is defined as the covariance of the two variables divided by the product of their standard deviations: $r=\frac{\sum_{i=1}^{L}\left(x_{i}-\bar{x})(y_{i}-\bar{y}\right)}{\sqrt{\sum_{i=1}^{L}{\left(x_{i}-\bar{x}\right)}^{2}}\sqrt{\sum_{i=1}^{L}{\left(y_{i}-\bar{y}\right)}^{2}}}$. When computing the sample Pearson correlation, *r*, at site *v*, *X* denotes the site’s vector of observed genotype dosages and *Y* denotes the site’s vector of imputed genotype dosages. The sample Pearson correlation is calculated with the assumption that the genotype dosages are accurately estimated. The sample Pearson correlation is a function of two vectors, both of length *L*. The value of *L* varies across sites for two reasons: the random nature of the masking scheme and non-uniform representation of sites within the set of validation genotypes defined by Caller A and B. The Pearson correlation coefficient is undefined when either *L*<2 or when the vector of imputed genotype dosages is invariant.

To calculate imputation accuracy, we masked a set of validation genotype dosages, imputed, and calculated the sample Pearson correlation between observed and imputed genotype dosages. We employed two different methods, Caller A and Caller B, to define the set of validation genotypes for cross-validation experiments. Caller A returns a genotype dosage for individual *d* at site *v* if individual *d* was surveyed a minimum of seven times at site *v* and returns NA otherwise. The second method, Caller B, returns a genotype dosage for individual *d* at site *v* if the most likely genotype is at least 10 times more likely than the second most likely genotype and returns NA otherwise. We found that cross-validation experiments using Caller A and B validation genotypes returned similar results for imputation scenario 1 (data not shown), resulting in our decision to run scenario 2 and 3 using only Caller B validation genotypes.

We simulated a scenario where genotypes were missing in a random fashion across the genome and obtained estimates of imputation accuracy using 10-fold cross validation. The masking scheme is best visualized by describing the datasets as matrices, where the rows represent biallelic sites and the columns represent individuals. The elements in a matrix represent genotypes: individual *d* has genotype $G_{d}^{\left(v\right)}=g$ at marker *v*. We extracted each genotype’s read depth from the VCF file using VCFtools [18]. We partitioned the set of validation genotypes into 10 equally sized, disjoint subsets: *M1*, *M2*, …, *M10*. Each subset corresponds to a fold in the 10-fold cross-validation scheme. As an example, we generated the masked dataset for fold 1 by taking the original data matrix, finding the coordinates of the genotypes belonging to the set *M1*, and setting the elements in these coordinates to missing. This masking scheme resulted in 10 masked datasets (i.e. 10 folds). We calculated the imputation accuracy on a per-site basis for each fold and the imputation accuracy on a per-individual basis for each fold. We then calculated the median imputation accuracy per-marker and the median imputation accuracy per-individual across the 10 folds.

### Measuring computation cost

We measured computation time as the number of CPU minutes required to complete the imputation of one dataset. All jobs were submitted to the Computational Biology Service Unit at Cornell University, which uses an eight core Linux (Centos 6.2) Dell PowerEdge M600 with 16GB RAM.

## Results

### Imputation with No Reference Panel

We imputed masked genotypes at 20205 SNPs on chromosome 5 in a sample of 694 individuals from the GG population. In this section, we report the results from cross-validation experiments where the set of validation genotype dosages was defined using Caller B (see Methods).

**Fig 1:**
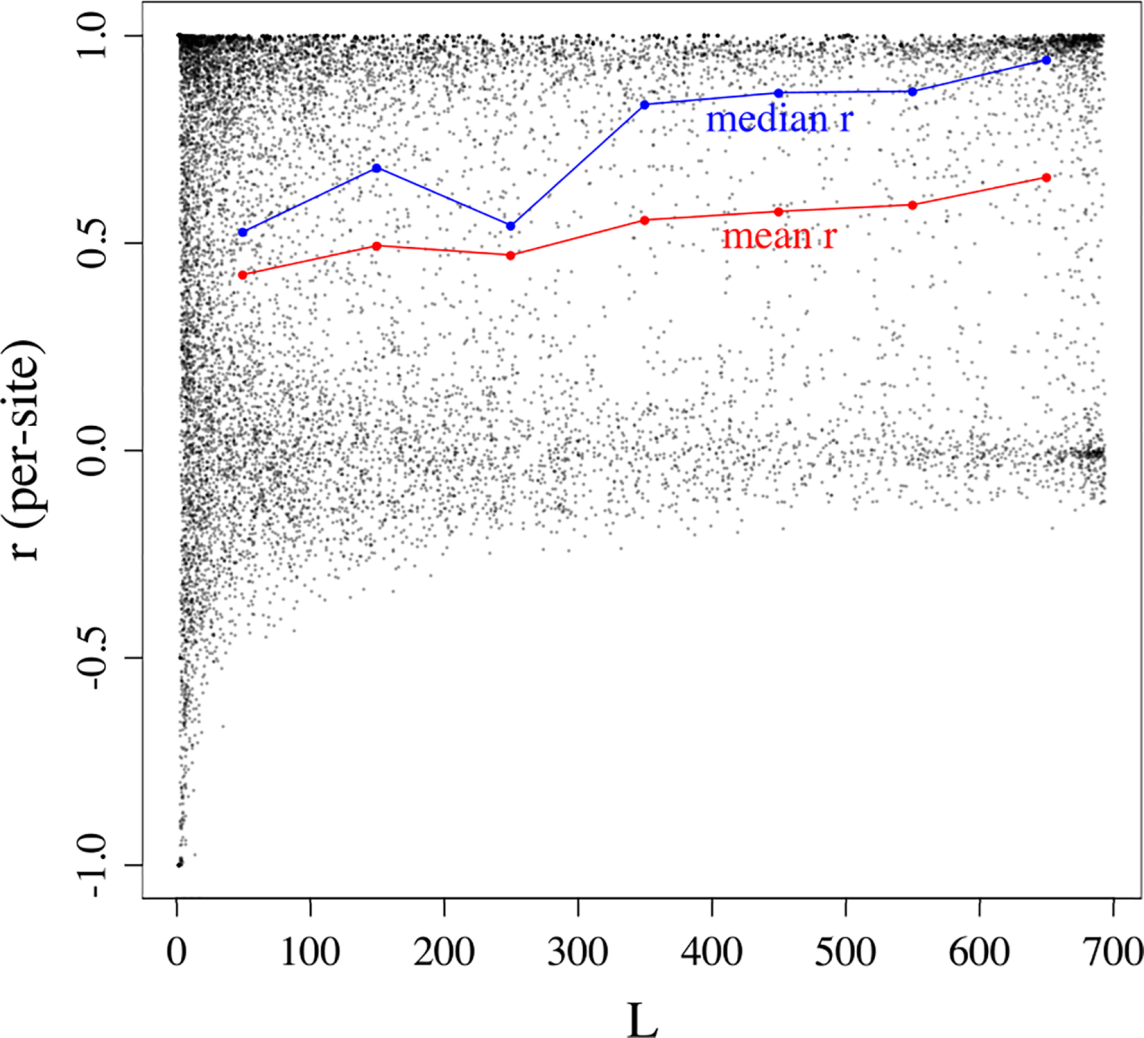
Estimates of accuracy as a function of *L* for 13028 sites imputed with Beagle. Imputation accuracies were estimated using the sample Pearson correlation coefficient, *r*. The sample Pearson correlation is a function of two vectors, both of length *L*. Fig 1. presents estimated accuracy as a function of *L* for set A sites (*n* = 13028). The range of *L* is divided into a series of seven equally sized bins (i.e. *0 < L ≤ 100*, *100 < L ≤ 200*, *…*, *600 < L ≤ 700*). Accuracy estimates were divided into bins according to their corresponding values of *L*. Bin means and medians are presented as red and blue points, respectively.

The sample Pearson correlation is a function of two vectors, both of length *L*. The value of *L* varies across sites and individuals because genotype masking occurs at random and because genotype call rates vary across the 20205 sites (see Methods). The sample correlation coefficient at site *v* is undefined under two scenarios: when *L*<2 (true for 34 of the 20205 sites in the dataset) and when the vector of imputed genotype dosages at site *v* has a variance equal to zero. The latter occurs when imputation returns identical genotype dosages for all *L* masked genotypes at site *v*. We obtained accuracy estimates for Beagle at 13028 sites (set A) and 19933 sites (set B) for glmnet. Set A is a subset of B, i.e. every member of set A is also a member of set B. Fig 1 presents estimated accuracy as a function of *L* for sites in set A imputed with Beagle. As might be expected, we observed greatest variation among accuracy estimates for small *L* (Fig 1). We removed sites with *L*<30 from our analysis, leaving us with 9737 sites (set C) to analyze. We selected a filter threshold of 30 somewhat arbitrarily but opted for a moderate-stringency filter to avoid removing a large subset of sites from our analysis.

**Fig 2:**
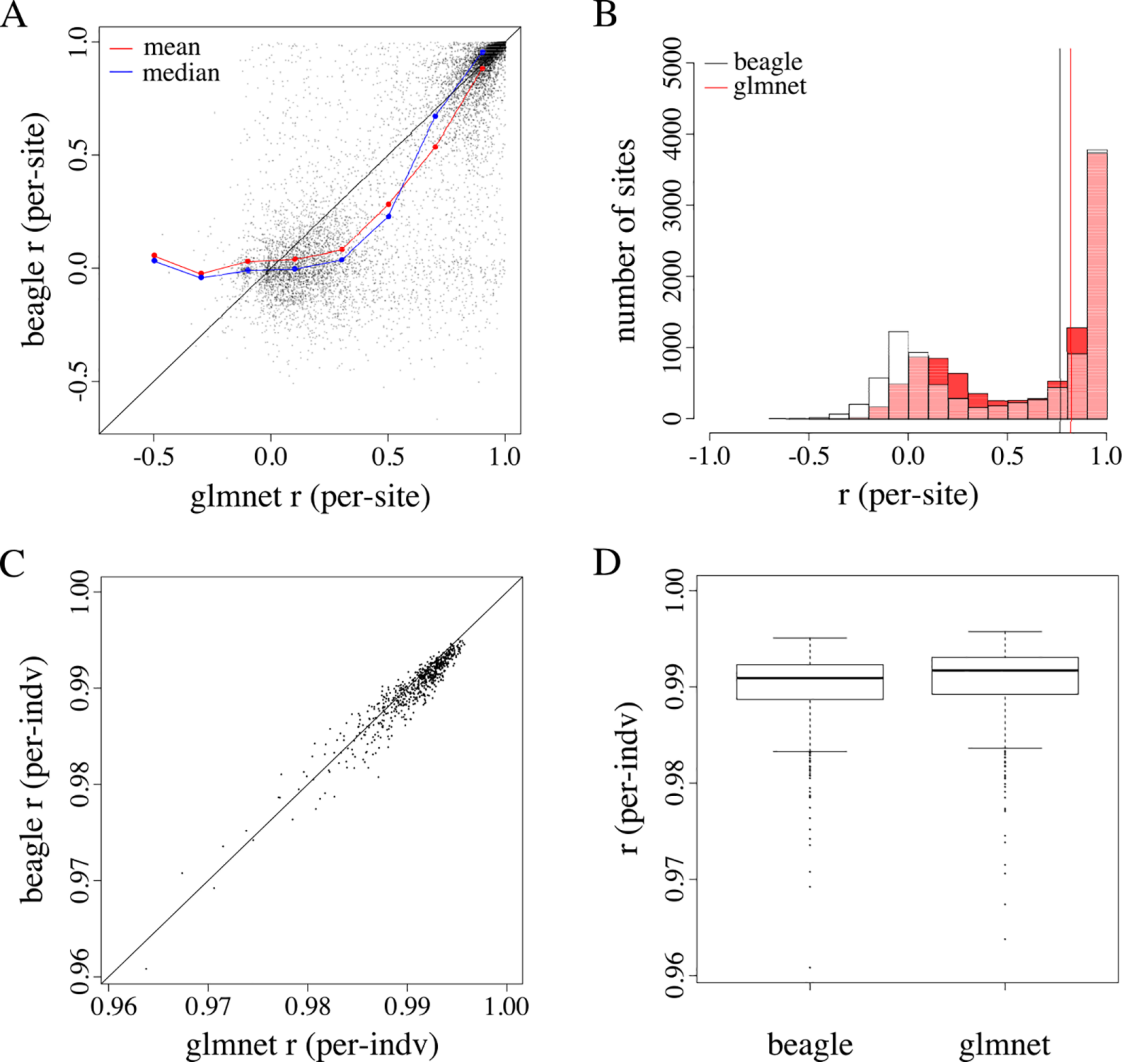
A summary and comparison of per-site and per-individual imputation accuracy from Beagle and glmnet imputation. (A and B) The *x*- and *y*-axes report estimates of imputation accuracy for glmnet and Beagle, respectively. Each point represents the estimated accuracy for a single site (A) and individual (B). (C) Both Beagle and glmnet produced bimodal distributions of per-site accuracies, with median per-site imputation accuracies of 0.76 (black vertical line) and 0.82 (red vertical line), respectively. (D) Both methods produced left-skewed distributions of per-individual accuracies, with median per-individual accuracies of 0.991 and 0.992 for Beagle and glmnet, respectively.

Fig 2 summarizes and compares the accuracy of Beagle and glmnet imputation at the site and individual level. Both Beagle and glmnet produced bimodal distributions of per-site accuracies, with median per-site imputation accuracies of 0.76 and 0.82, respectively (Fig 2B). We argue that this bimodality results from an overrepresentation of low-frequency variants, a hallmark of HTS-derived datasets. Both methods produced left-skewed distributions of per-individual Pearson correlations, with nearly identical medians (0.991 and 0.992 for Beagle and glmnet, respectively; Fig 2D).

### Proportion of missing data and read depth

**Fig 3:**
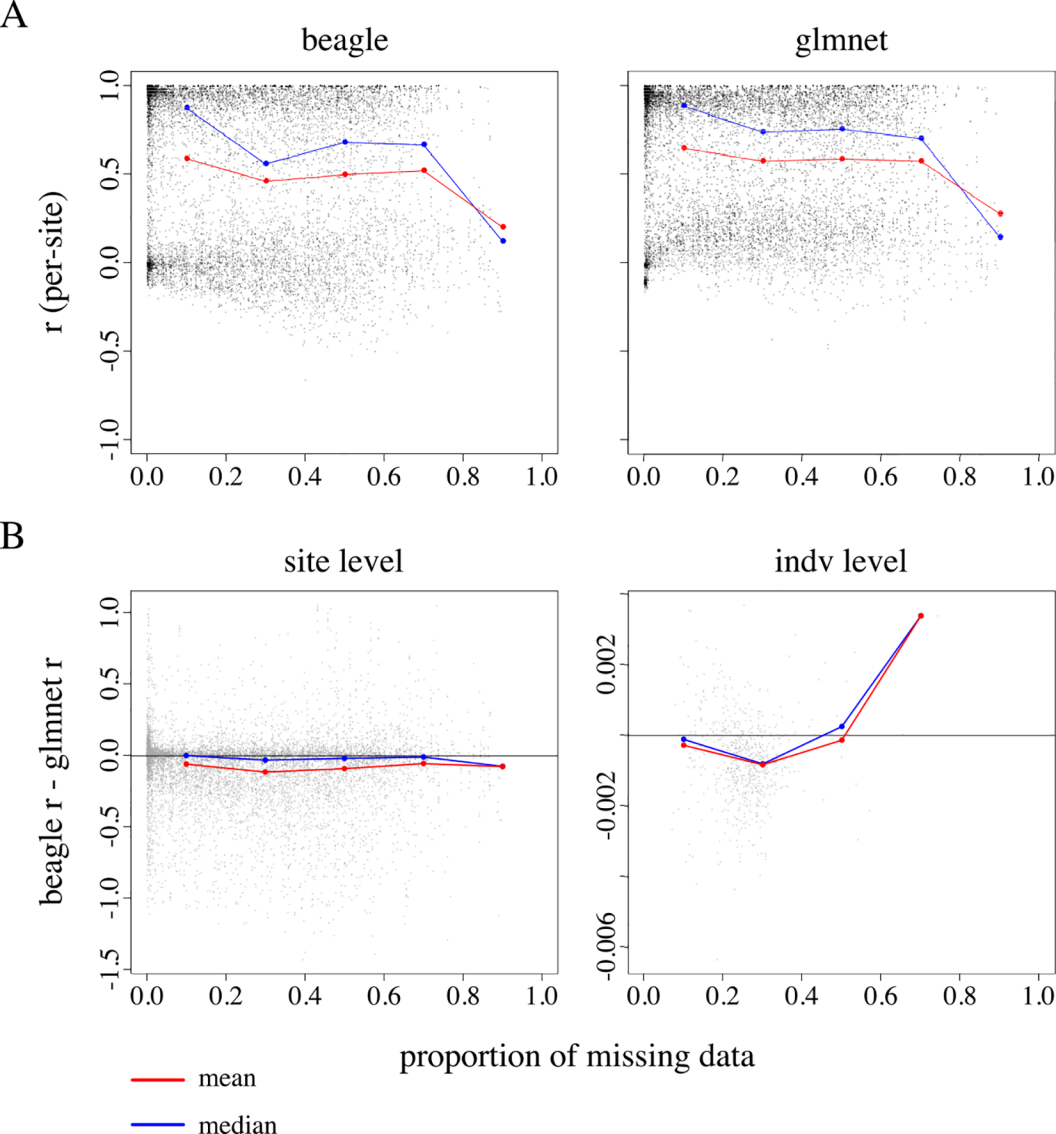
Per-site and per-individual imputation accuracy as a function of missing data and median read depth. (A) Beagle and glmnet imputation accuracy as a function of missing data for sites in set C (*n* = 9737). (B) The *x*- and *y*-axis display the proportion of missing data and the accuracy difference between Beagle and glmnet at the site and individual level. The range of *x* is divided into ten-equally sized bins (i.e. *0*.*00 < x ≤ 0*.*10*, *0*.*10 < x ≤ 0*.*20*, *…*, *0*.*90 < x ≤ 1*.*00*), and accuracy differences are divided into bins according to levels of missing data. Bin means and medians, summarizing the data within each bin, are displayed as red and blue points, respectively. Points falling on the black vertical line at *y* = 0 indicate no observed accuracy difference between Beagle and glmnet imputation. Points falling below *y* = 0 represent cases where glmnet imputes with higher accuracy relative to Beagle.

We examined the effect of the proportion of missing data on imputation accuracy at the site and individual level (Fig 3). As might be expected, we observed a decline in imputation accuracy as the level of missing data increased. Beagle appears to show greater sensitivity to levels of missing data relative to glmnet, particularly when the proportion of missing data at a site falls within the (0.1, 0.5] interval (Fig 3A and 3B). We observed essentially no difference between the two imputation methods when examining accuracy at the individual level (Fig 3B).

### Minor allele frequency

**Fig 4:**
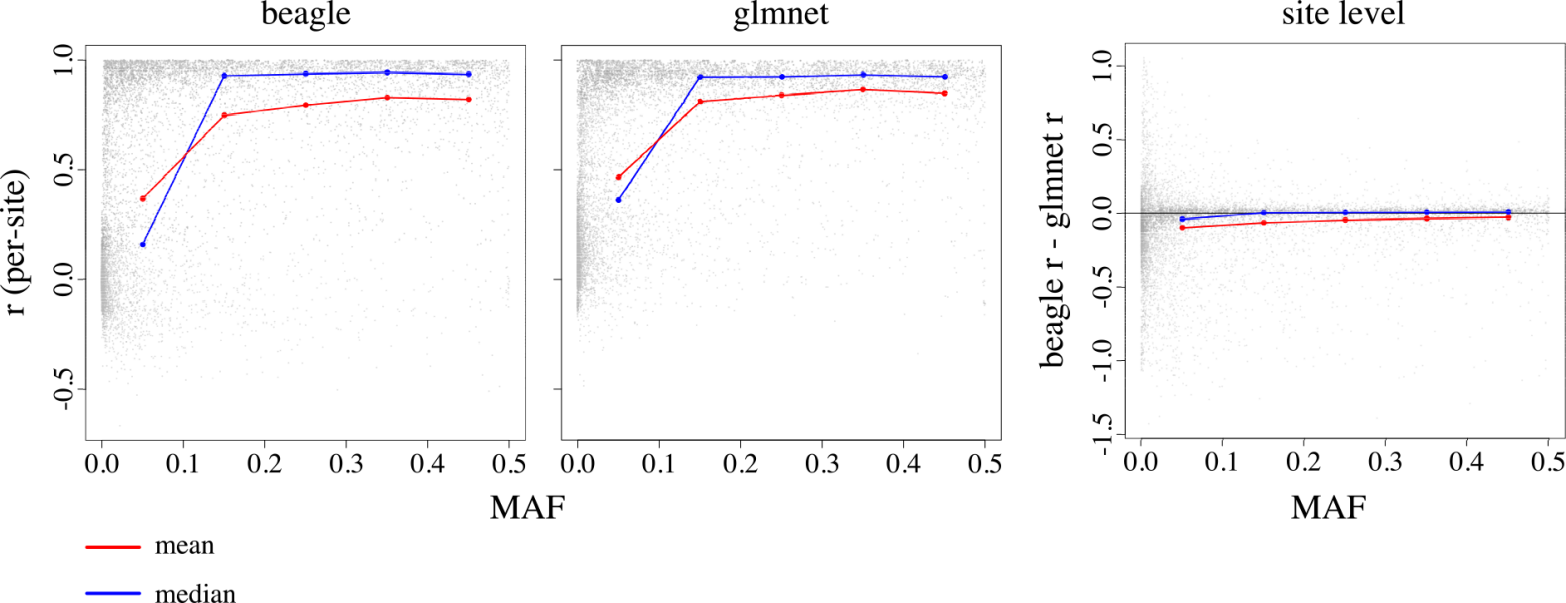
Imputation accuracy as a function of MAF. The left and middle panels show per-site accuracy of Beagle and glmnet as a function of (estimated) MAF. The right-most panel shows the difference in accuracy between Beagle and glmnet at each site as a function of MAF. We observed the greatest difference in accuracy at low-frequency variants. Low-frequency variants were imputed with high variance.

We estimated the minor allele frequency (MAF; the minor allele at a site could be either the reference or alternative allele listed in the VCF file) at all 20205 sites using the sample of 694 individuals from the GG population. Fig 4 presents per-site *r* as a function of estimated MAF for the 9737 sites in set C. We divided the range of *x* into five-equally sized bins (i.e. *0*.*00 < x ≤ 0*.*10*, *0*.*10 < x ≤ 0*.*20*, *…*, *0*.*40 < x ≤ 0*.*50*), and summarized accuracy values within each frequency bin using the mean and median (Fig 4). We observed a decrease in accuracy as MAF decreased and greatest variance in low-frequency bins (Fig 4 left and middle panel). These two trends are consistent with previous results suggesting that sites harboring rare alleles are more difficult to impute accurately relative to sites harboring more common alleles [3]. Glmnet appears to impute with slightly higher accuracy than Beagle at all MAF bins (Fig 4 right panel).

### Reference Panel Size and Composition

**Fig 5:**
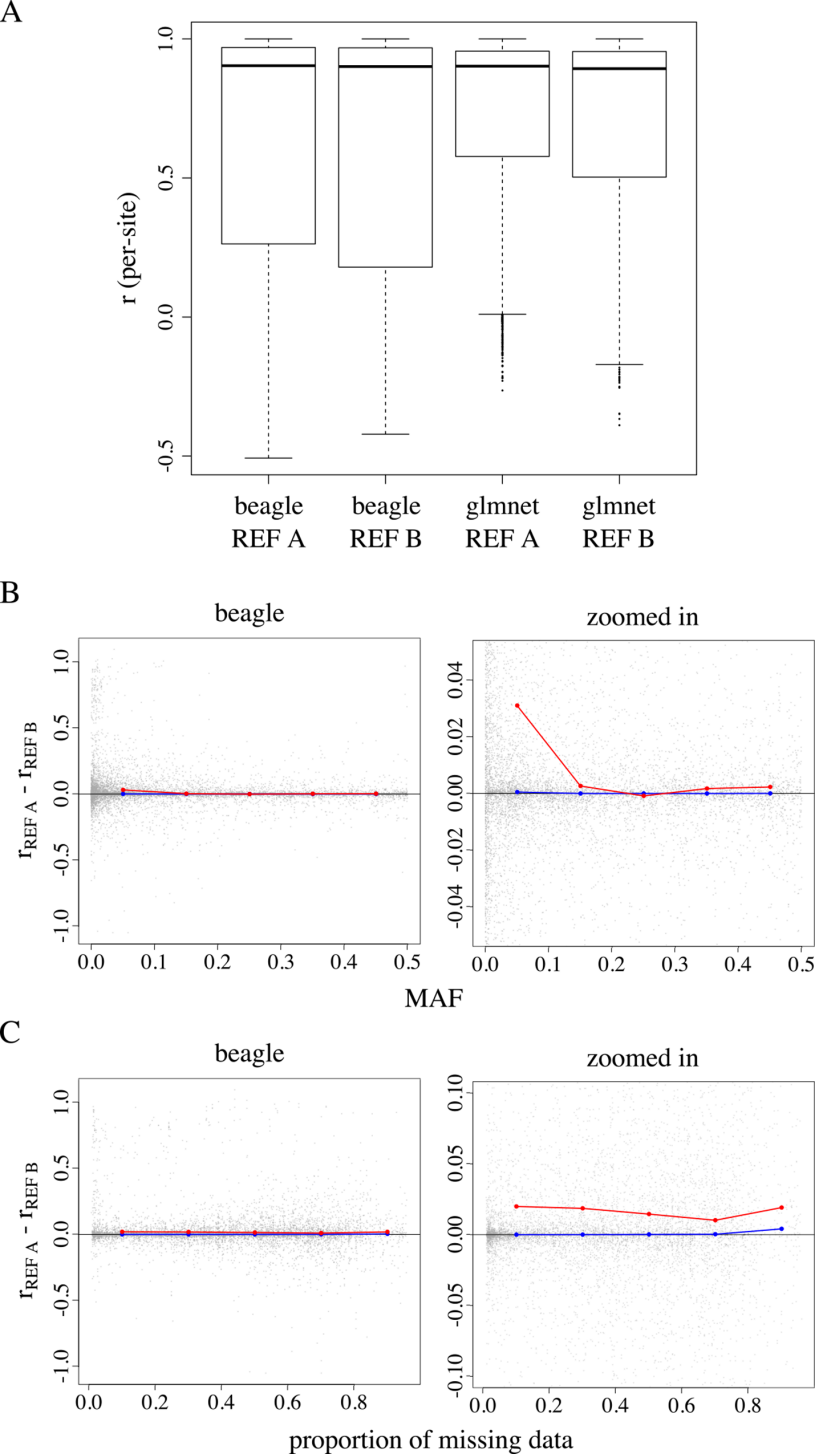
**The accuracy difference between reference panel A and panel B as a function of MAF and proportion of missing data for 11535 sites.** (A) Genotypes in a sample of 2490 C1 individuals were imputed using two different reference panels: reference panel A, comprised of 694 phased GG individuals, and reference panel B, comprised of 80 phased individuals listed as progenitors of the C1 population. (B and C) Points falling on the black vertical line at *y* = 0 indicate no observed accuracy difference when imputing with reference panel A or B. Points falling below *y* = 0 represent cases where Beagle imputes with higher accuracy when using reference panel B relative to imputing with reference panel A.

We next investigated the effect of reference panel composition on imputation accuracy (Fig 5). Fig 5 summarizes Beagle and glmnet imputation accuracy in a sample of 2490 individuals from the C1 population for genotypes imputed with a reference panel of 694 and 80 individuals (Fig 5A). [19] reported considerable increases in Beagle’s imputation accuracy with increased reference panel size across all minor allele frequencies, with the greatest increase at low-frequency variants. We, however, observed essentially no difference in the median per-marker *r* when imputing with the larger reference panel (Fig 5A). Sites with a MAF ≤ 0.01 appeared to benefit the most when imputing with a larger reference panel, but gains in accuracy were small (Fig 5B). We observed modest gains in mean accuracy across all levels of missing data when imputing with the larger reference panel (Fig 5C). Overall, Beagle and glmnet imputed missing genotype with similar accuracies regardless of the reference panel used. Beagle required a slightly longer runtime relative to glmnet (Table 1).

**Table 1:** A summary of Beagle and glmnet’s computation cost (in seconds) and median per-site and per-individual accuracy under scenario 1, 2, and 3.

	Mean run time (seconds)		Median per-site *r* (%)		Median per-individual *r* (%)	
	Beagle	Glmnet	Beagle	Glmnet	Beagle	Glmnet
**Scenario 1**	2249.6	12477.86	76.48	81.94	99.17	99.09
**Scenario 2**	63713.5	56295.45	90.37	90.21	99.34	99.30
**Scenario 3**	43935.4	34551.17	90.05	89.31	99.36	99.29

(Top) We calculated the mean computation time across the 10 folds of each cross-validation experiment. (Middle) We calculated the median *r* across sites and reported this as a scalar summary of imputation accuracy in that cross-validation experiment. (Bottom) We calculated the median *r* across individuals and reported this as a scalar summary of imputation accuracy in that cross-validation experiment

## Discussion

Imputation accuracy was calculated as the correlation between the observed genotype dosage (estimated from allelic count data in the AD subfield of the VCF file) and the imputed genotype dosage. We note that to obtain true measures of imputation accuracy, the imputed genotype dosage should be correlated with the true genotype, rather than the observed genotype dosage. Unfortunately, true genotypes are not known and observed genotype dosages must be used instead. The accuracy based on correlation to the observed genotype dosages under-estimates the true imputation accuracy in two ways. First, there is error associated with the observed genotype dosage (resulting from sequencing errors, alignment errors, etc.) that reduces the correlation. Second, the observed genotype dosage of individual *i*, at site *j* were computed using one source of information—the observed sequence data from individual *i*, at site j. The imputed genotype dosage from Beagle and glmnet, in contrast, were computed using a multi-sample LD approach. Multi-sample LD methods infer the genotype dosage of individual *i*, at site *j* by jointly analyzing data from multiple individuals in the sample, at site *j* and at nearby sites (i.e. information regarding LD). The use of information from multiple individuals and patterns of LD has been shown to lead to significant improvements in genotype-calling accuracy for low-depth sequence data (for an example, see [20]).

Using a set of validation genotypes at biallelic SNPs on chromosome 5, we found that Beagle and glmnet impute missing variants with similar accuracies. When comparing the two methods at the site level, glmnet appears to impute with (moderately) higher accuracy relative to Beagle, regardless of levels of missing data. We, however, observe little difference between the two methods when measuring accuracy at the individual level (Fig 3B). We observed the greatest difference in accuracy between the two methods in scenario 1 (imputation guided by no reference panel). Differences, however, were only moderate, suggesting that 1) human-tailored imputation algorithms can produce relatively accurate genotype estimates when applied to datasets derived from non-human organisms and 2) these algorithms, which were developed and optimized to impute ascertained variants, appear amenable for imputation of variants discovered via an HTS methods such as GBS.

The unique aspects of the datasets derived from a non-human organism, such as cassava, and HTS methods, such as GBS, do not affect Beagle’s imputation accuracy in ways we do not understand or expect. For instance, we observed a decrease in imputation accuracy as MAF decreased (Fig 4 left), consistent with previous results suggesting that sites harboring rare alleles are more difficult to impute accurately relative to sites harboring more common alleles [3]. Results also indicate that the Beagle algorithm is robust to deviations from the HWE assumption that underlies the Beagle algorithm. HWE is violated in domesticated species, which have undergone generations of controlled mating and directional selection.

The modest difference in imputation accuracy between Beagle and glmnet was in some ways unexpected, largely because the two algorithms employ contrastingly different approaches to modeling genotype data. Glmnet does not attempt to directly relate observed correlation patterns to any underlying biological process, whereas Beagle specifies a statistical model for the biological aspect of the problem–namely, the haplotypes that generated the observed LD structure. Both algorithms leverage data at a subset of markers to impute missing genotypes at a particular locus, but they employ very different subset selection strategies. Glmnet selects markers solely on measures of pairwise correlation, ignoring locus order and spacing. Beagle, in contrast, focuses on a small number of nearby markers when imputing missing genotypes at a particular site (localized haplotype-cluster model). Correlation between markers is a localized phenomenon; that is, there tends to be less LD between loci that are far apart than between loci that are close together. The apparent correlations observed between distant markers are largely statistical artifacts, i.e. noise introduced by sampling variation. While glmnet and Beagle produced similar results in our cross-validation experiments, we reason that there are situations in which Beagle will outperform glmnet (e.g. when levels of spurious associations between distant markers is high relative to true levels of LD). In addition to decreased sensitivity to spurious associations between distant markers, probabilistic, phasing methods, such as Beagle, offer additional benefits, such as providing phased haplotypes and measures of imputation accuracy estimated from posterior genotype probabilities.

In scenario 2 and 3, we used a sample of 2490 C1 individuals to compare the accuracy of genotype imputation with a cosmopolitan reference panel (reference panel A) and a best-match panel (reference panel B). Reference panel A consists of 694 individuals, a subset of who are list as C1 parents in pedigree records (*n* = 78). Reference panel B, in contrast, consists entirely of individuals listed as C1 parents in pedigree records (*n* = 80). The set of C1 parents in panel A is a subset of panel B. We found that imputation using reference panel A and B resulted largely in similar imputation accuracies across sites. We find this reassuring for two reasons: 1) the 617*2 haplotypes from the non-parental individuals in reference panel A appear to serve as good proxies for the haplotypes of the two C1 parents that are present in panel B but absent in panel A and 2) adding ‘extraneous’ haplotypes to the reference panel appears to introduce little error to the imputation procedure, consistent with previous observations made by those in the human genetics community [3]. Imputation with reference panel A required more computation time relative to imputation with panel B (by approximately 1.5X). In practice, however, the task of constructing a best-match reference panel is considerably more challenging and computationally expensive than the one presented here. We reason that a cosmopolitan reference panel is a good fallback choice when the optimal panel composition is unclear and if one has the computational resources to employ a large reference panel for imputation.

## Supporting Information

S1 Fig **Description of reference panel A and B and the three imputation scenarios.** (A) The Venn diagram shows the composition of reference panel A and B. (B) We evaluated Beagle and glmnet under three imputation scenarios: imputation guided by no reference panel (left), a reference panel with large genetic diversity (reference panel A; middle), and 3) a reference panel that closely matches the ancestry of the study sample (reference panel B; right).
(PDF)

S2 Fig Distribution of variants across chromosome 5. The white and red histogram displays the distribution of all variant sites (30018) and biallelic SNPs (20302) along the length of chromosome 5, respectively.
(PDF)

S3 Fig No evidence of population structure among the 696 reference panel individuals. No records of genetic relatedness among the 696 reference panel individuals exist. We, therefore, performed a PCA to explore whether there is any evidence of population structure among reference panel individuals. Reference panel individuals contributing zero offspring to the C1 population appear as grey dots. Reference panel individuals contributing >0 offspring to the C1 population appear as red dots with diameters scaled proportionally to the number of offspring contributed by the individual.
(PDF)

S4 Fig Distribution of the proportion of missing data per biallelic SNP. The proportion of missing data at a given site is measured across the 694 GG individuals. The term “missing” denotes zero reads observed at a given site for a given individual. We removed sites with >90% missing data, leaving a total of 20205 sites for cross-validation experiment 1. We used this same set of sites for scenarios 2 and 3 for reasons given in the main text.
(PDF)

S5 Fig Distribution of the mean read depth per site. (A) The histogram shows the distribution of the mean read depth per site averaged across all 694 GG individuals. (B) The histogram shows the distribution of the mean read depth per site averaged across all 696 reference panel individuals. The red vertical line marks the mean of the distribution.
(PDF)

S1 Table Members of the GG population. (XLSX)

S2 Table Pedigree records for C1 population. (XLSX)
