# Supplementary material for: Evaluating Imputation Algorithms for Low-Depth Genotyping-By-Sequencing (GBS) Data
Source: PLoS One. 2016 Aug 18;11(8):e0160733. doi: 10.1371/journal.pone.0160733 (PMC4990193; doi:10.1371/journal.pone.0160733)
Supplement: S1 Fig — Description of reference panel A and B and the three imputation scenarios. (A) The Venn diagram shows the composition of reference panel A and B. (B) We evaluated Beagle and glmnet under three imputation scenarios: imputation guided by no reference panel (left), a reference panel with large genetic diversity (reference panel A; middle), and 3) a reference panel that closely matches the ancestry of the study sample (reference panel B; right). (PDF) [file pone.0160733.s001.pdf]

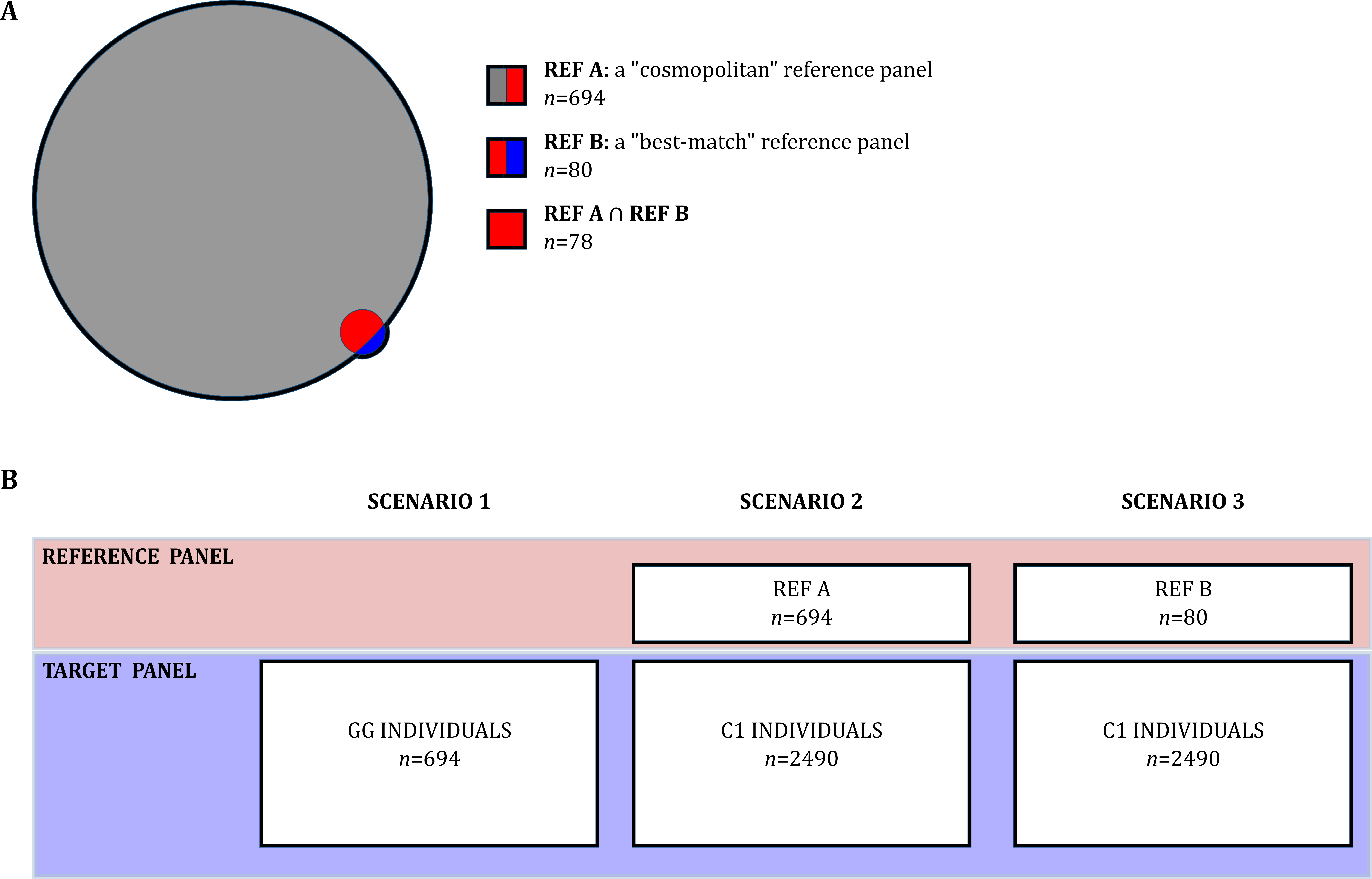
A
REF A: a "cosmopolitan" reference panel
n=694
REF B: a "best-match" reference panel
n=80
REF A ∩ REF B
n=78
B
SCENARIO 1
SCENARIO 2
SCENARIO 3
REFERENCE PANEL
REF A
n=694
REF B
n=80
TARGET PANEL
GG INDIVIDUALS
n=694
C1 INDIVIDUALS
n=2490
C1 INDIVIDUALS
n=2490
