# Supplementary figures and images for: Evaluating Imputation Algorithms for Low-Depth Genotyping-By-Sequencing (GBS) Data
Source: PLoS One. 2016 Aug 18;11(8):e0160733. doi: 10.1371/journal.pone.0160733 (PMC4990193; doi:10.1371/journal.pone.0160733)

## distribution of variants across chromosome 5

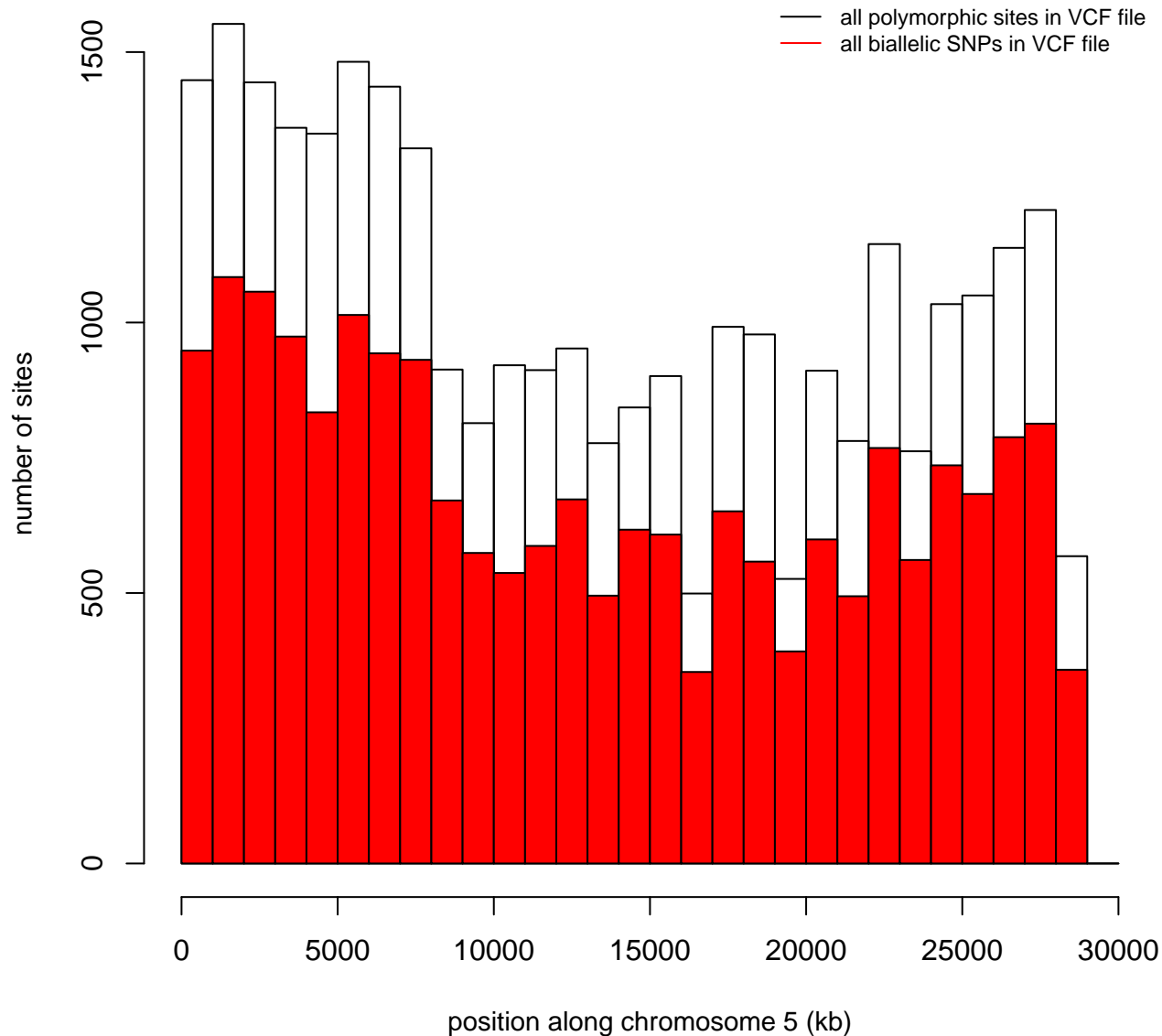

Supplement: S2 Fig — The white and red histogram displays the distribution of all variant sites (30018) and biallelic SNPs (20302) along the length of chromosome 5, respectively. (PDF) [file pone.0160733.s002.pdf]

EM imputation

— non C1 parent  
— C1 parent

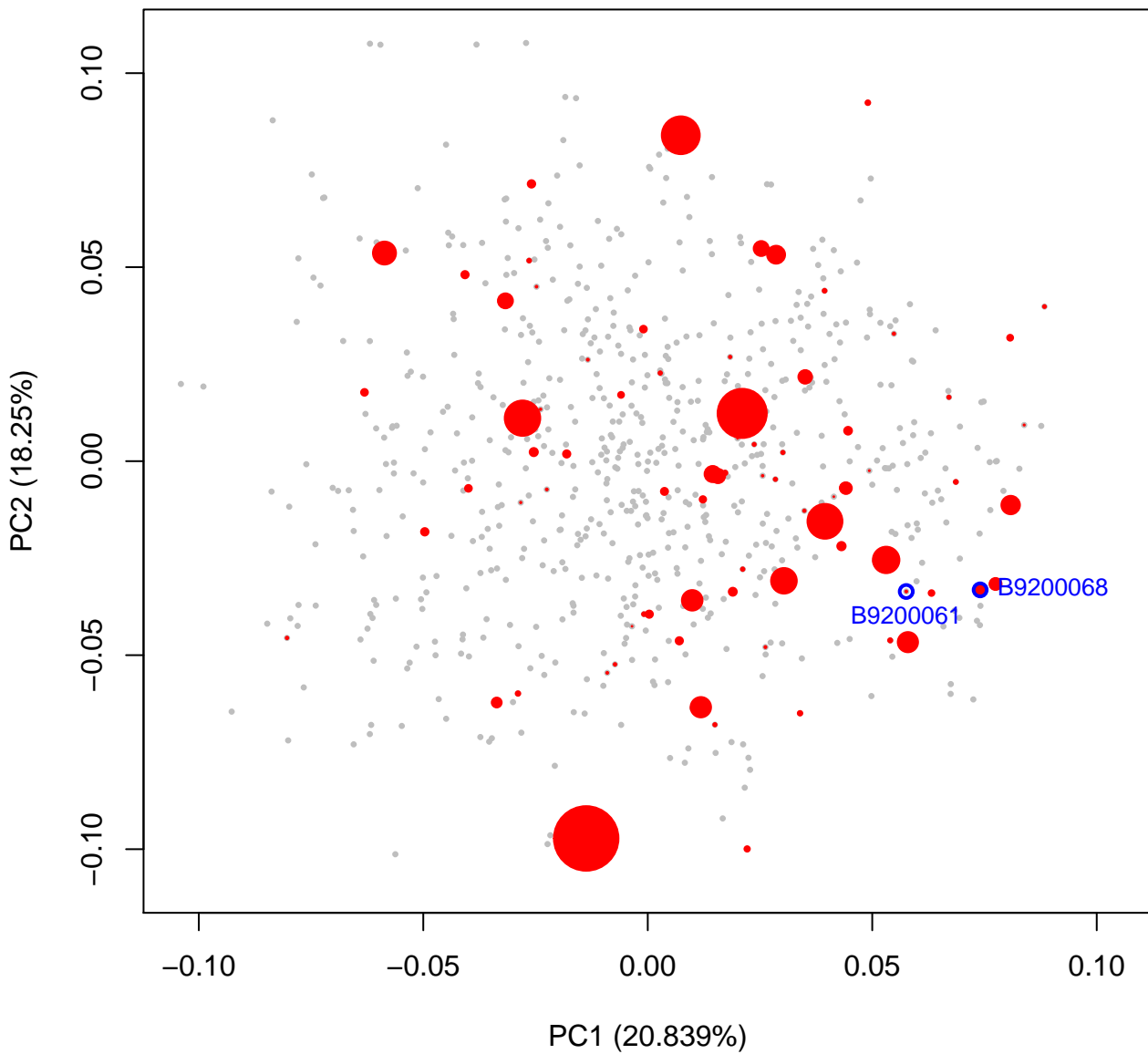

Supplement: S3 Fig — No records of genetic relatedness among the 696 reference panel individuals exist. We, therefore, performed a PCA to explore whether there is any evidence of population structure among reference panel individuals. Reference panel individuals contributing zero offspring to the C1 population appear as grey dots. Reference panel individuals contributing >0 offspring to the C1 population appear as red dots with diameters scaled proportionally to the number of offspring contributed by the individual. (PDF) [file pone.0160733.s003.pdf]

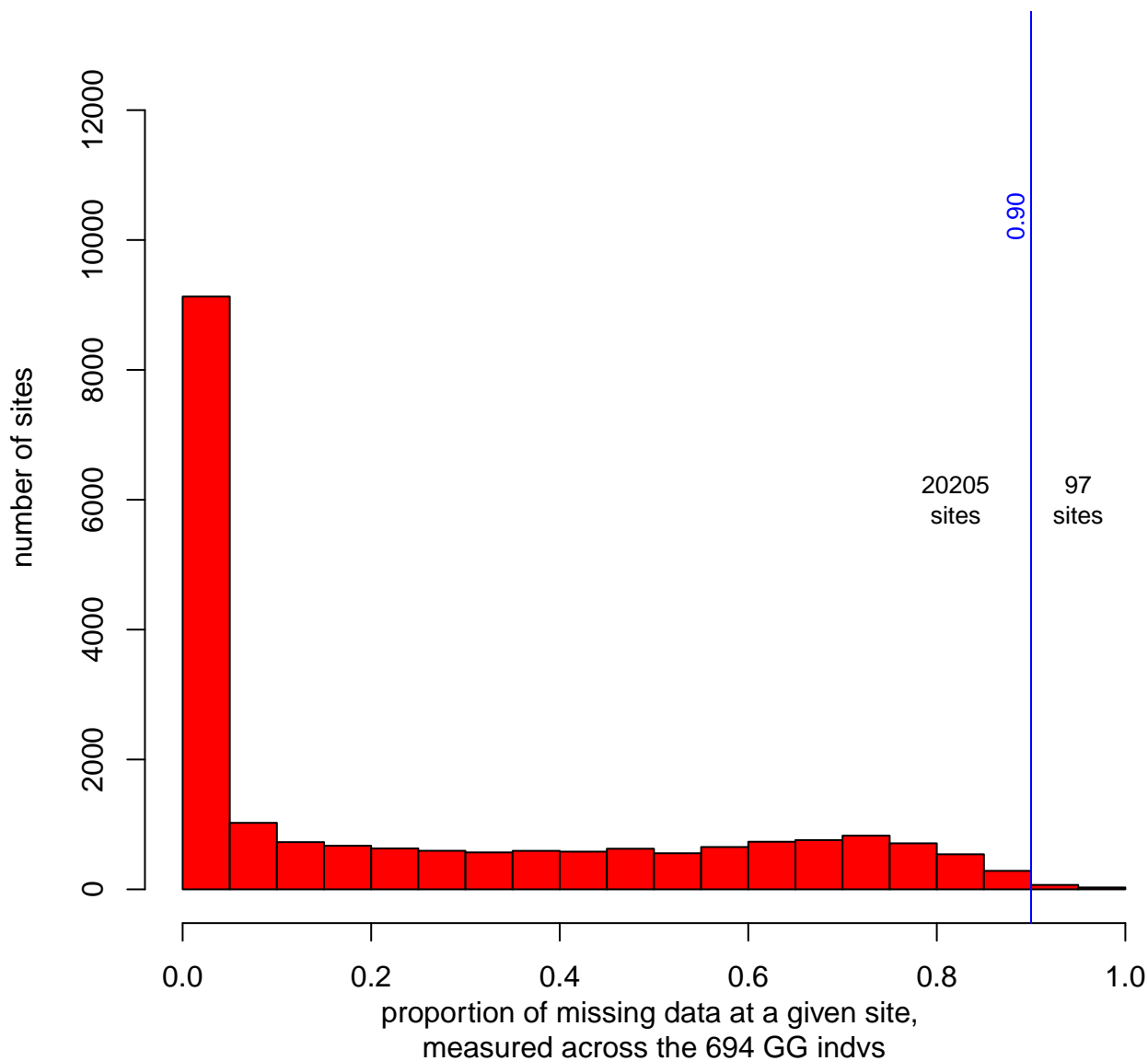

Supplement: S4 Fig — The proportion of missing data at a given site is measured across the 694 GG individuals. The term “missing” denotes zero reads observed at a given site for a given individual. We removed sites with >90% missing data, leaving a total of 20205 sites for cross-validation experiment 1. We used this same set of sites for scenarios 2 and 3 for reasons given in the main text. (PDF) [file pone.0160733.s004.pdf]

A

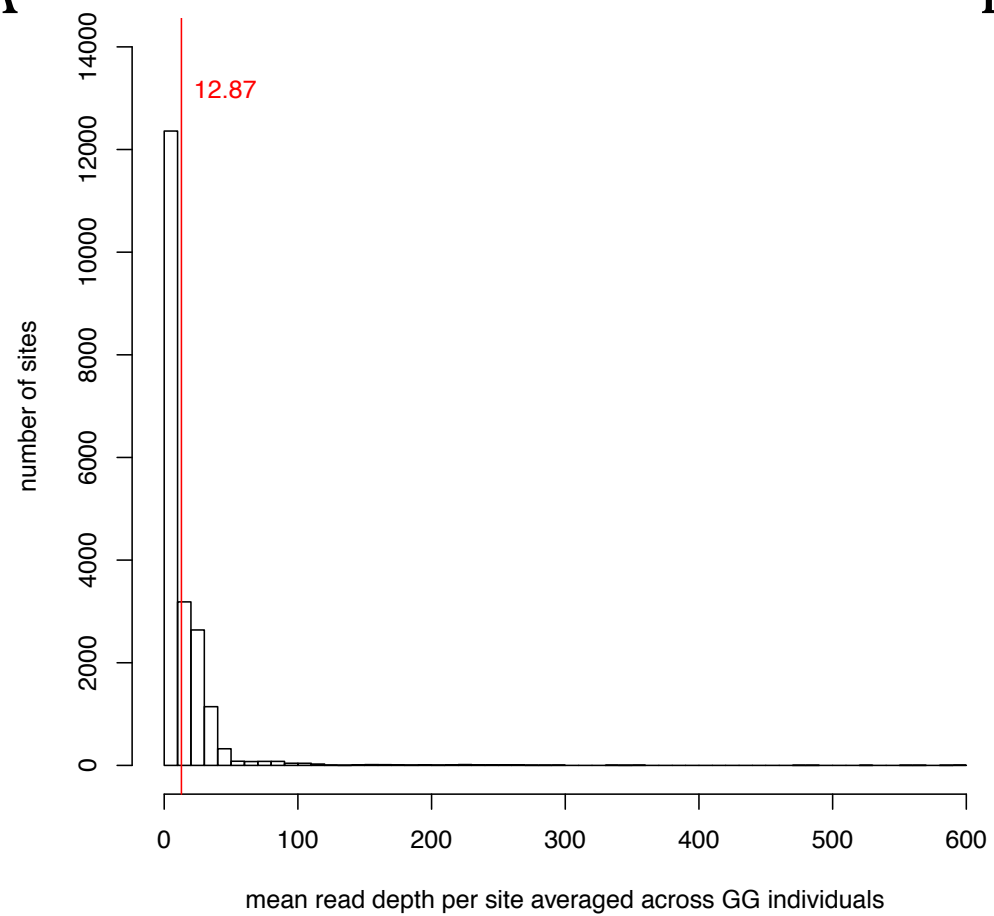

B

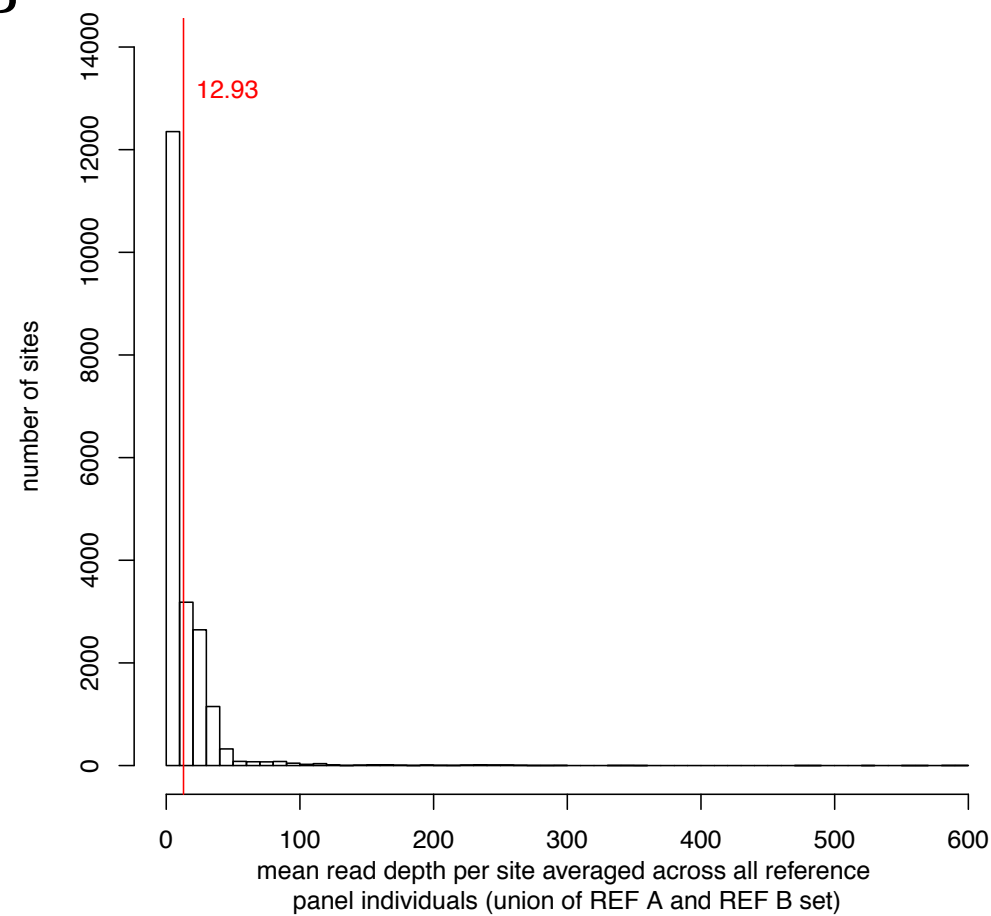

Supplement: S5 Fig — (A) The histogram shows the distribution of the mean read depth per site averaged across all 694 GG individuals. (B) The histogram shows the distribution of the mean read depth per site averaged across all 696 reference panel individuals. The red vertical line marks the mean of the distribution. (PDF) [file pone.0160733.s005.pdf]
